# Supplementary material for: Identification of photoperiod-induced specific miRNAs in the adrenal glands of Sunite sheep (Ovis aries)
Source: Front Vet Sci. 2022 Jul 22;9:888207. doi: 10.3389/fvets.2022.888207 (PMC9354845; doi:10.3389/fvets.2022.888207)
Supplement: Supplementary file 1 [file Data_Sheet_1.ZIP › Supplementary file/miRDeep2 file.zip!/miRDeep2 file/Know_Structure/SP42A1/oar-mir-30b.pdf]

miRBase precursor : oar-mir-30b
Total read count : 22218
oar-miR-30b read count : 22048
remaining reads : 170

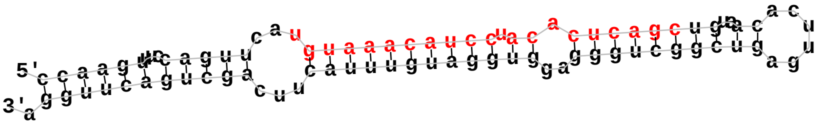

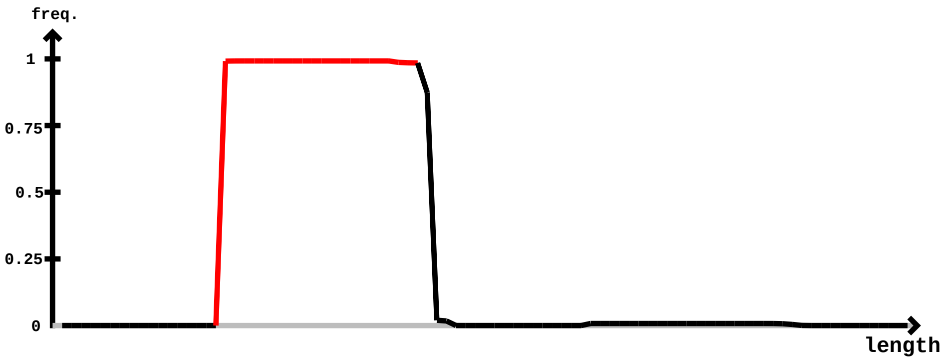


```
                              oar-miR-30b
5'- ccaaguuuccaguucauguaaacauccuacacucagcuguaacacuugagucggcugggagguggauguuuacuucagcugacuugga   -3'    exp
    ((((((...(((((...((((((((((.((.((((((((..((......))))))))))..))))))))))))...))))))))))).   reads      mm        sample
    ...............auguaaacauccuacacucagcu..................................................   1          0         seq
    ................uguaaacauccuaUacu.......................................................   1          1         seq
    ................uguaaacauccuacacu.......................................................   1          0         seq
    ................uguGaacauccuacacuc......................................................   2          1         seq
    ................uguaaacauccuacacAc......................................................   1          1         seq
    ................uguaaacauccuacacuc......................................................   79         0         seq
    ................uguaaacauccuacacuU......................................................   36         1         seq
    ................uguaaacauccuacacuca.....................................................   35         0         seq
    ................uguaaacauccuacacucag....................................................   13         0         seq
    ................uguaaacauccuacacucagc...................................................   2459       0         seq
    ................uguaaacauccuacacucagcu..................................................   18965      0         seq
    ................uguaaacauccuacacucagcug.................................................   54         0         seq
    ................uguaaacauccuacacucagcugu................................................   381        0         seq
    ................uguaaacauccuacacucagcugua...............................................   3          0         seq
    .................guaaacauccuacacucagc...................................................   1          0         seq
    .................guaaacauccuacacucagcu..................................................   12         0         seq
    .................guaaacauccuacacucagcug.................................................   1          0         seq
    ..................uaaacauccuacacucagcu..................................................   2          0         seq
    ...................aaacauccuacacucagcu..................................................   1          0         seq
    ......................................................cugggagguggauguuuac...............   2          0         seq
    ......................................................cugggagguggauguuuacu..............   17         0         seq
    ......................................................cugggagguggauguuuacuu.............   57         0         seq
    ......................................................cugggagguggauguuuacuuc............   83         0         seq
    ......................................................cugggagguggauguuuacuuca...........   11         0         seq
```
